# Attentional modulation as a mechanism for enhanced facial emotion discrimination: The case of action video game players

**DOI:** 10.3758/s13415-022-01055-3

**Published:** 2023-01-20

**Authors:** Alina Ciobanu, Kengo Shibata, Lna Ali, Kenneth Rioja, Søren K. Andersen, Daphne Bavelier, Benoit Bediou

**Affiliations:** 1 Faculty of Psychology and Educational Sciences, University of Geneva, Geneva, Switzerland; 2 Campus Biotech, University of Geneva, Geneva, Switzerland; 3 Nuffield Department of Clinical Neurosciences, University of Oxford, Oxford, UK; 4 School of Psychology, University of Aberdeen, Aberdeen, UK; 5 Department of Psychology, University of Southern Denmark, Odense, Denmark

**Keywords:** Action video games, Attention, Emotion processing, SSVEP

## Abstract

Action video game players (AVGPs) outperform nonvideo game players (NVGPs) on a wide variety of attentional tasks, mediating benefits to perceptual and cognitive decision processes. A key issue in the literature is the extent to which such benefits transfer beyond cognition. Using steady-state visual evoked potentials (SSVEP) as a neural measure of attentional resource allocation, we investigated whether the attentional benefit of AVGPs generalizes to the processing of rapidly presented facial emotions. AVGPs (*n* = 36) and NVGPs (*n* = 32) performed a novel, attention-demanding emotion discrimination task, requiring the identification of a target emotion in one of two laterally presented streams of emotional faces. The emotional faces flickered at either 2.0 Hz or 2.5 Hz. AVGPs outperformed NVGPs at detecting the target emotions regardless of the type of emotion. Correspondingly, attentional modulation of the SSVEP at parieto-occipital recording sites was larger in AVGPs compared with NVGPs. This difference appeared to be driven by a larger response to attended information, as opposed to a reduced response to irrelevant distractor information. Exploratory analyses confirmed that this novel paradigm elicited the expected pattern of event-related potentials associated with target detection and error processing. These components did not, however, differ between groups. Overall, the results indicate enhanced discrimination of facial emotions in AVGPs arising from enhanced attentional processing of emotional information. This presents evidence for the attentional advantage of AVGPs to extend beyond perceptual and cognitive processes.

**Supplementary Information:**

The online version contains supplementary material available at 10.3758/s13415-022-01055-3.

## Introduction

The ability to focus on relevant information whilst resisting distractions is a core cognitive process underlying various aspects of our everyday behavior: from perception to cognition. A growing body of literature suggests that this process of attentional control can be enhanced by playing action video games (AVG), specifically first- or third-person shooter games (Bavelier and Green, 2019; see Dale et al., 2020 for further characterization of action-like video games). These video games require dynamic processing of rapidly displayed content whilst ignoring distractions. Attention needs to be divided and distributed between objects that appear unpredictably in the periphery and also flexibly shifted to maintain focus on high-resolution tasks, such as aiming and shooting. Additional demands to switch between goals under time constraints are also present. Therefore, attentional control is critical and potentially trainable through AVG play. Furthermore, regular AVG players (AVGPs) have shown superior cognitive performance that generalizes beyond the game setting. This aligns with the reported enhancements in the top-down allocation of attentional resources (Bediou et al., 2018). The ubiquity of this video game genre and the reported positive effects on attentional control, and in turn cognition, have raised several fundamental questions about the extent to which such benefits generalize beyond cognition. Recent reports have shown far transfer of action gaming benefits to learning novel tasks, likely achieved through faster learning of perceptual templates allowing task-relevant features to be processed with priority (Bejjanki et al., 2014; Zhang et al., 2021). Game-related perceptual gains have also been shown to enhance initial acquisition of scene context memories (Zinchenko et al., 2022). We asked whether the reported attentional enhancement generalizes to the attentional processing of socioaffective emotional stimuli as well.

The benefits of AVG play have been established using various attention-demanding tasks. AVGPs outperform Non-Video Game Players (NVGPs) in multiple object tracking (Green and Bavelier, 2003, 2006a), visual search (Hubert-Wallander et al., 2011; Wu and Spence, 2013), and attention-in-time tasks, such as attentional blink (Dale and Green, 2017; Green and Bavelier, 2003; Wong and Chang, 2018). Furthermore, enhanced top-down attentional control in AVGPs facilitates the identification of peripheral targets among distractors (Chisholm et al., 2010; Chisholm and Kingstone, 2012; Green and Bavelier, 2006b), the processing of irrelevant flankers at low perceptual loads (Dye et al., 2009), and the division of attention across locations (Green and Bavelier, 2006a; West et al., 2008). These behavioral benefits have been linked to functional reconfigurations of attentional networks in AVGPs (Föcker et al., 2018). In AVGPs, there is less recruitment of the frontoparietal network as task demands increase (Bavelier et al., 2012), indicating that for simple tasks, this network is recruited more efficiently in AVGPs compared with NVGPs. This pattern of reduced activation is also observed in individuals with high cognitive flexibility (Armbruster et al., 2012) and with repeated practice or performance improvements (Beauchamp et al., 2003; Landau et al., 2004; Schneiders et al., 2012; Yotsumoto et al., 2008).

Further studies providing evidence for optimized resource allocation in AVGPs has used electroencephalography (EEG) to assess neural processes that are associated with behavior. Presenting stimuli at a fixed frequency generates periodic EEG responses at corresponding frequencies in the form of steady-state visual evoked potentials (SSVEPs) (Regan, 1966). This approach can be used to separate neural responses to attended targets from neural responses to irrelevant distractors (Krishnan et al., 2013; Mishra et al., 2011; Wu et al., 2012). Mishra et al. (2011) used a fast-paced visual attention task and found that AVGPs outperform NVGPs at detecting oddball digits embedded in a sequence of letters, all presented in peripheral vision, whilst fixating at a central fixation cross. This behavioral advantage was related to enhanced attentional modulation in AVGPs compared with NVGPs. Upon closer inspection, distractor suppression, rather than an increased neural activity to relevant stimuli was found to drive the attentional difference between groups. The comparison between action (First Person Shooter (FPS)) and nonaction (Role Playing Game (RPG)) players yielded similar results, wherein FPS players outperformed RPG players in a rapid visual search with varying numbers of locations to attend to or ignore (Krishnan et al., 2013). Performance was related to the suppression of noise rather than an enhanced signal to relevant stimuli. A causal effect of AVG play for enhanced top-down spatial selective attention by increased inhibition of distractors was also reported in a 10-hour video game intervention study (Wu et al., 2012). Taken together, AVGPs appear to have improved attentional resource allocation compared to NVGPs, mostly through enhanced suppression of irrelevant or distracting information.

These attentional advantages of AVGPs have predominantly been documented using low-level non-emotional stimuli such as letters, digits, and shapes (see meta-analysis, Bediou et al., 2018). The stimuli we process in daily life are much more complex and contextual. Emotional faces are one such example that may require different processing demands. There has been a rich debate about the extent to which emotional faces require attention to process (Pessoa et al., 2002; but see also Vuilleumier, 2005). The present study circumvents this debate by investigating the benefits of AVGPs in a difficult peripheral emotion discrimination task that is, by nature, highly attention-demanding. Below, we review the literature examining the relationship between video game experience and emotion processing focusing on action video games (which often contain violent content) and violent video games (which may or may not implement action mechanics and thus do not necessarily fall under the action video game genre). Notably, all these studies used low-attention demanding tasks, unlike the tasks used in this study.

Past studies examining facial expression recognition in action and violent video game players have generated mixed results. One study found that players of violent games showed impaired recognition of disgust, compared with gamers who did not play violent games and also displayed an increased accuracy and faster recognition of fearful emotions (Diaz et al., 2016). This may arise from an in-game advantage of sensitivity to fearful stimuli. In contrast, negative associations between violent game exposure and the recognition of negative emotions (measured with facial expression matching) were found in both adolescents and adults, after controlling for age, gender, and trait empathy (Miedzobrodzka et al., 2021). The Facial Expressions Matching Test used in this study required participants to perceptually match an emotional expression from a choice of three emotional expressions, removing the need to identify or label the emotions. When extending the question from violent games to action games, no difference in emotion recognition was found between AVGP and NGVP in several standard 2-AFC recognition tasks (Pichon et al., 2021). Furthermore, using the technique of reverse inference, no group difference was observed in the mental representations of emotions. In other words, the spatial and temporal patterns of facial muscle activation—also called action units (AUs)—that support emotion recognition were comparable in AVGP and NVGP. Therefore, reports on the perceptual advantages of AVGPs extending to the processing of socially relevant stimuli such as facial emotions remain inconclusive.

Three other studies have addressed the causality of video game play on facial emotion processing. First, the effect of a short-term (15-minute) acute exposure to violent gameplay on an emotion detection task was investigated (Kirsh and Mounts, 2007). Participants were shown faces changing from neutral to emotional (happy or angry) and tasked to press a key as soon as they recognized the emotion. The RT difference between happy and angry faces was reduced after playing 15 minutes of a violent game (compared with 15 min of a nonviolent game). This result was interpreted as evidence for a reduced happy face advantage following violent gameplay, although it is unclear whether the effect was driven by slower recognition of happiness, faster recognition of anger, or a combination of both. No difference in emotion recognition accuracy was reported. In contrast, a more recent study showed slower identification of angry faces relative to happy faces (resulting in an increased happy face advantage) after 25 minutes of exposure to violent video games compared with nonviolent games (Liu et al., 2017), thus reporting an opposite pattern of short-term effects. Lastly, the causal long-term effect of action gameplay was studied using an emotion search task with schematic emotional faces (angry and happy in neutral or vice versa) before and after 10 hours of action or non-action video gameplay (Bailey and West, 2013). No group differences were reported (only the no-contact group was slower at detecting neutral targets). However, a complex pattern of EEG results suggested an improved ability to detect facial emotions in the AVG-trained group, which was characterized by differences in ERP amplitudes related to the allocation of attention to positive facial expressions. In sum, the evidence for the effect of AVG play on the processing of facial emotions remains unclear and has been mostly carried out using emotion recognition tasks with limited attentional demands. Given our goal of studying the effects of attention on facial emotion processing in AVGPs, we employed a cross-sectional approach contrasting AVGPs and NVGPs as a method to maximize the possibility of finding group differences.

To specifically address whether AVGP’s attentional advantages generalize to the processing of emotional stimuli, we administered a novel attention-demanding emotion discrimination task whilst measuring SSVEPs. As alluded to previously, SSVEPs characterize oscillatory signals of cortical neurons in response to stimuli flickering at a regular temporal frequency. This provides a sensitive neural measure of the allocation of processing resources (Andersen and Muller, 2010; Muller et al., 2008) as attended (relevant) and unattended (irrelevant) stimuli can be quantified using SSVEP amplitudes tagged to specific visual frequencies (e.g., Davidson et al., 2020; Toffanin et al., 2009). The attention-demanding emotion discrimination task was inspired by the previously mentioned study investigating resource allocation in AVGPs using letters and digits (Mishra et al., 2011). An emotion-dependent version of this task was developed where participants were presented with two peripheral streams of emotional faces and were asked to detect rare target emotions in the cued stream. The irrelevant stream had to be ignored. The targets were fewer than the nontargets, unpredictable, and embedded in nontargets, which also were emotional faces, limiting the pop-out effect of targets. Mapping between targets and nontargets was counterbalanced across blocks. Two emotions were used as either targets (e.g., happiness, surprise) or nontargets (e.g., disgust, anger) to limit the possibility that task performance would be driven by perceptual cues (e.g., white areas of the eyes or the degree of mouth opening) instead of emotional processing. Half of the blocks consisted of rare positive valence targets (happy/surprise) among more common negative valence faces (disgust/anger), and the other half consisted of rare negative valence targets (disgust/anger) among more common positive valence faces (happy/surprise). The task was attentionally demanding as: 1) the two streams of facial emotions were presented simultaneously on the left and right of a central fixation cross; 2) participants were required to maintain fixation on the central cross (verified with EOG); and 3) participants were cued to attend one of the streams and discriminate infrequent emotional targets (e.g., happy, surprised) embedded with more frequent emotional distractors (e.g., angry, disgusted). To the best of our knowledge, this is the first SSVEP study using peripheral facial expressions, because faces are highly complex stimuli. Each stream flickered at low frequencies of either 2.0 Hz or 2.5 Hz. Before this SSVEP task, participants also were asked to evaluate each emotional face in a separate emotional recognition task. For a subset of participants, the Multiple Object Tracking (for a review, see Meyerhoff et al., 2017) and the Useful Field of View tasks were administered as measures of attentional control.

While AVGPs and NVGPs were expected to have comparable judgments of facial emotion when viewing faces in isolation, we hypothesized that the greater attentional control of AVGPs would predict an enhanced attentional modulation in AVGPs compared with NVGPs.

## Materials and methods

### Participants

The final sample size of 68 participants included 36 AVGPs (5 females; age: mean [*M*] = 23.25, standard deviation [*SD*] = 4.11) and 32 NVGPs (2 females; age: *M* = 23.19, *SD* = 4.27). Groups did not differ in age, *t*(66) = 0.06, *p* = 0.95 nor in gender, *t*(66) = 1.03, *p* = 0.31.

We initially recruited 40 AVGPs and 47 NVGPs with normal or corrected-to-normal vision based on responses to the Bavelier Lab Video Game Questionnaire (https://www.unige.ch/fapse/brainlearning/vgq/) completed at most 12 months before participation in the study. The questionnaire assessed current and past video gameplay usage across different gaming genres. The exact questionnaire and its scoring can be found on the Open Science Framework project registration (Shibata et al., 2020). Briefly, participants who reported playing 5 hours or more of first- and/or third-person shooter gameplay experience per week were classified as AVGPs. In addition, experience with action-RPG/adventure, Sports/Driving, and Real-Time Strategy/Multiplayer Online Battle Arena also contributed to an AVGP classification. Participants were considered as NVGPs if they satisfied the two following criteria: 1) played less than 1 hour per week in each of the following game genres - action (First- and Third-person Shooter) and action-like video games (action Role Playing Game/Adventure, Sports/Driving games, and Real-Time Strategy/Multiplayer Online Battle Arena); 2) played less than 3 hours a week in any of the following game genres - non-action Role Playing Game, Turn-based Strategy games, Music games or any other game genres. Participants were not considered for the study if they qualified as high media multitaskers with a Media Multitasking Index (MMI) score equal to or superior to 5.9 (Ophir et al., 2009), presented neurological problems, or had a history of psychiatric disorders (e.g., schizophrenia or depression). Three NVGPs were excluded as their responses to the questionnaires were outdated and no longer reflected their status at the time of testing. One AVGP was excluded due to technical issues during data collection. Ten participants (3 AVGPs / 7 NVGPs) were removed due to poor EEG signal quality during data collection. Five NVGPs were also excluded due to excessive artifacts found during the cleaning of raw EEG data.

This study was run in two distinct data collection phases, first with 29 participants (15 AVGPs / 14 NVGPs) and second with 39 participants (21 AVGPs / 18 NVGPs). After the first phase, we confirmed that expected neural amplitudes could be extracted from SSVEPs with our novel attention-demanding emotion discrimination task. The study was pre-registered on OSF before the start of the second phase of data collection (Shibata et al., 2020). Power analysis indicated that a target sample size of 64 (32 per group) was required to achieve 80% power to detect an effect size of hedge’s g = 0.63 (as per the effect size reported for behavioral performance difference between AVGP and NVGP for top-down attention by Bediou et al., 2018), with an alpha threshold of .05 using a one-tailed independent-sample *t*-test. The lead experimenter in data collection was fully blinded to the participants’ group allocations. Procedures were approved by the University of Geneva Ethics Review Board, and all participants provided written, informed consent before data collection. All participants were compensated at a rate of 30 CHF per hour for their participation.

### Experimental tasks

#### Apparatus

The emotion recognition task and the SSVEP emotion discrimination task were programmed and administered through Presentation (Neuro Behavioral Systems, Version 18). The Multiple Object Tracking (MOT) and Useful Field of View (UFOV) tasks were programmed in Javascript and administered through a web browser and saved locally on a MySQL database. All tasks were run under Windows 7 operating system using a high-performance linearized LCD glass monitor (22.5 inches adjustable ViewPixx monitor – 1,920 x 1,080 pixels, 120 Hz). Participants were tested in a dark room with a viewing distance of 57 cm from the monitor, enforced using an adjustable chin and forehead rest.

EEG recordings were made from 64 Ag/AgCl electrodes placed following the 10-20 international system (BioSemi Active Two amplifier system). We used a modified configuration of electrodes with 4 electrodes from central-frontal areas moved to lower parieto-occipital sites to increase spatial resolution over lateral occipital-temporal sites (Adamian et al., 2019; Antonov et al., 2020). In addition, 4 external EOG electrodes were used to monitor lateral and vertical eye movements.

#### Emotion recognition task

Distinct stimuli consisting of 24 individual identities (12 females, 12 males) from the Karolinska Directed Emotional Faces database (Lundqvist, Flykt, and Öhman, 1998) were used. Each stimulus consisted of a face expressing either happiness, surprise, anger, or disgust. The identities of the faces were selected such that the perceived normative recognition rates were comparable across different emotions. To control for the variability of shape, size, and hair of the identities, an oval contour mask was placed around each stimulus. Physical characteristics were controlled by removing all remaining hair and teeth using the photo-editing software Gimp. Gray-scaled images were normalized for contrast and brightness so that all stimuli had a Gaussian distribution with an equivalent mean and standard deviation. Participants were asked to distinguish the emotion and rate the intensity and valence of 96 faces presented individually at the center of the screen. Emotions were identified using a key press of the corresponding list of emotions (“happiness,” “surprise,” “anger,” “disgust,” “fear,” and “other”). The rating for the valence and intensity of each stimulus was carried out using a computer mouse on a 5-point scale from 1 (very negative/low intensity) to 5 (very positive/high intensity). No time limit was enforced on this task.

#### Attention-demanding emotion discrimination task

**Fig. 1:**
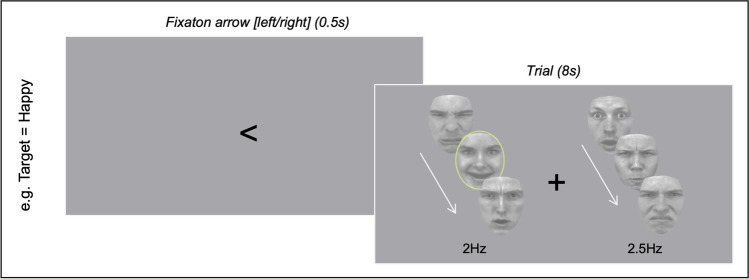
Trial design of attention-demanding emotion discrimination task. Following a fixation arrow indicating which stream (left or right) to attended to, participants were presented with an 8 second trial of two streams of emotional stimuli presented concurrently. Participants were required to detect specific target emotions in the relevant stream indicated by the initial arrow (</>). Targets could appear on either side and were embedded in other emotional stimuli (as shown in the green circled target - not shown during the experiment). Each of the two streams of faces were presented at either 2Hz or 2.5Hz (but never the same between streams). Participants were required to hold their gaze at the central fixation throughout the trial and response as fast and as accurately as possible

This task was a modified version of the attentional task implemented in Mishra et al. (2011). The emotional stimuli were the same as the ones used for the emotion recognition task. A black fixation cross was centrally presented for 2.5 s before the trial onset. An arrow indicating the cued (to-be-attended) side was displayed for 0.5 s. Two streams of faces were presented for 8 s, simultaneously on the left and right sides of the cross, centered at 5.3 degrees eccentricity. Faces were displayed at a frequency of either at 2.0 Hz or 2.5 Hz, such that each face was presented for either 500 ms (at 2.0 Hz) or 400 ms (at 2.5 Hz) without an interstimulus interval. The stimulus frequencies used in this study are lower than that of previous studies using nonemotional stimuli (8.6 Hz and 12.0 Hz) (Mishra et al., 2011; Morgan et al., 1996) to account for the time required to process laterally presented emotional faces in an attentionally demanding task. The presentation speed, as well as the attended side, varied across trials. The number of targets within one 8 s trial varied randomly between 0 and 4. The first target appeared at the earliest, 400 ms after the onset of the sequence. The minimum delay between two consecutive targets was set to 1 s. There were no repetitions of an identical face within a given trial. A 1 s response window was allowed for stimulus-response (Fig. 1).

Participants were asked to press the spacebar each time they identified a target emotion in the cued/attended stream of faces. The streams consisted of either rare instances of angry/disgusted faces among happy/surprised faces or happy/surprised faces among angry/disgusted faces. The two streams had an equivalent valence of emotions (negative or positive) with the same number of rare targets. Before the start of each of the 8 s trials, an arrow pointing to the left or right indicated the stream to attend to. The participants were asked to visually fixate on the central fixation cross throughout each of the 8 s trials. A total of 320 trials were distributed over 16 sessions. The procedure counterbalanced 8 (2x2x2) different conditions: 1) attended side (left or right); 2) frequency assignment (2.0 Hz on the left and 2.5 Hz on the right side or vice versa); and 3) the emotion of target faces (fear/disgust or happy/surprise) presented in pseudo-randomized order. The target emotions were separated into two distinct and counterbalanced sessions. Each session contained 8 blocks of 20 trials. The attended side and frequency assignments were pseudo-randomized within each block of 20 trials. Feedback on percentage correct and the number of times a response was made was displayed at the end of each block. Each session started with practice trials for the corresponding target emotion; the experimenters confirmed task comprehension and gaze fixation. Auditory feedback was provided for every response made during the practice session. The dependent variables for this task were response accuracy and the SSVEP amplitudes obtained from the EEG recordings.

#### Multiple Object Tracking (MOT)

The Multiple Object Tracking (MOT) task used parameters employed in previous studies (Yung et al., 2015). Participants were tasked with tracking 1 to 5 moving targets among 16 moving stimuli. The stimuli with a 0.4-degree radius moved at 5 degrees per second in a 20-degree diameter area. All targets and distractors followed a random trajectory, with a 60% chance of changing direction by an angle drawn from a normal distribution (standard deviation of 12 degrees). Stimuli that collided with each other or with the perimeter reverted direction. Practice sessions had stimuli of 2 degrees per second and only 8 moving stimuli with visual feedback at every trial.

Targets were initially cued as blue sad faces (2D smileys) and distractors as yellow happy faces. Two seconds into the trial, the blue sad faces turned into yellow happy faces for another 4 s. Participants were required to track the initially cued blue faces throughout the trial. At the end of a trial, one of the stimuli was probed with a question mark. Participants were asked to indicate, using a keypress (B for blue, Y for yellow), whether the stimulus was initially a blue target or a yellow distractor. The task consisted of 45 trials in total, with 5 trials at 1 target and 10 trials at 2-5 targets administered in a pseudo-randomized order over 3 blocks of 15 trials. There were small breaks in between blocks, and percentage correct feedback was given at the end of each block. All participants started with a short practice session. The dependent measure was the percentage of correct responses. This task was administered before the EEG set up and only to participants recruited in the second phase of data collection.

#### Useful Field of View (UFOV)

The Useful Field of View (UFOV) task also used similar parameters as previously described (Yung et al., 2015). On each trial, participants viewed a central target—a 1° smiley with either short or long hair, and a peripheral target—a 1° star flashing at 4° eccentricity along one of 8 possible meridians (0°, 45°, 90°, 135°, 180°, 225°, 270°, or 315°). Each stimulus was followed by a visual noise mask (320 ms) on the whole screen. Participants were asked to report the position in which the peripheral target appeared and whether the central stimulus had long or short hair. Trials were considered correct when subjects accurately identified both the position of the peripheral target and the identity of the central target. A 3-up-1-down adaptive staircase procedure was used to change stimulus duration. Three consecutive correct answers led to a reduction in stimulus presentation duration by 1 frame (1/60 s) (2 frames for the first 3 reversals), whereas 1 incorrect answer led to an increase by 1 frame in stimulus presentation duration.

Subjects were tested in a single setting after administration of the MOT task. Participants first went through a list of visual instructions for the central and peripheral tasks and practiced conditions with both the central and peripheral targets. The task duration was dependent on the participant’s performance. The task ended when one of three conditions was met: 1) the staircase procedure reached 8 reversals; 2) participants completed 10 trials at the ceiling duration or floor duration; or 3) participants reached the maximum trial number of 72. The dependent variable was the detection threshold, calculated by averaging the duration of the stimulus presentation over the past 5 trials. Like the MOT task, this task was only administered to participants recruited for the second data collection phase.

#### EEG data processing

Pre-processing of EEG data was conducted using the EEGLAB toolbox and custom MATLAB scripts. Raw EEG data was first down-sampled to 256 Hz and pre-processed by removing linear drifts. The data was segmented into 9 s epochs, consisting of data from 1 s before stimulus onset and the 8 s of RSVP in a trial. A baseline correction between −1,000 ms and −500 ms also was applied to the data. We manually interpolated channels that were noted as frequently noisy during data collection and then executed the FASTER algorithm implemented in MATLAB for automatic artifact rejection (Nolan et al., 2010) using high-pass filtering at 1 Hz, low-pass filtering at 95 Hz, and notch filtering at 50 Hz. No ICA was used, although the algorithm removed noisy epochs. On average, FASTER rejected 8.97 epochs (±3.40), which did not differ between groups, *t*(66) = −0.36, *p* = 0.72. However, the signal quality for the right attended conditions had more noise and fewer epochs included in the analysis, *t*(67) = 2.97, *p* = 0.0042.

Lateral eye movements were recorded with a bipolar montage at the outer canthi of the eyes for horizontal EOG and above and below the eyes for vertical EOG. We manually checked every epoch for horizontal eye movements using EOG traces and removed epochs with significant drifts in eye movements by plotting average deflections on nonfiltered data. Trials with significant deflections, excessive blinking, or muscular activity were manually discarded. Entire epochs were removed if such events were found. The minimum number of trials required per condition was 12 and represented 30% of the maximum number of trials (i.e., 40) in each of the eight conditions (side [left/right] – presentation frequency [2/2.5 Hz] – target emotion [positive/negative target emotion]). Subjects were excluded if they had less than 30% of the epochs in a given condition. On this basis, five participants were excluded from further analysis, as reported in the participants’ section above. For the included participants, 22.94% of epochs were rejected. The proportion of rejections were not different between AVGPs and NVGPs, *t*(66) = −0.102, *p* = 0.919.

#### Data analysis

##### Behavior

In the emotion discrimination task, all responses between 200 ms and 1,000 ms after target onset were defined as hits. Outside these intervals, responses were categorized as false alarms. We computed the sensitivity index, d-prime (*d’*) by subtracting hit and false-alarm rates after transforming them with the inverse of the cumulative normal distribution:

$$d\text{'}=z(H)\hbox{--} z(FA)$$

False-alarm rates were computed using the approach by Bendixen and Andersen (2013) for continuous tasks:

$$FA=\frac{F}{\frac{TS}{TR}- NT}$$

Where F = number of false alarms, TS = total duration of trials (response window x 320 trials), TR = time window for which a correct response can be given (200 ms – 1,000 ms = 800 ms) and NT = total number of targets of TS. The hit rate was computed by dividing the correct hits by the number of trials. The response window was set to 8,800 ms, given that each trial is 8,000 ms, and the first 200 ms cannot be responded to, but responses were allowed 1,000 ms after the last stimulus. Mean response time was calculated in ms, including only correct responses or hits. The behavioral results only reflect performances in the attended stream. Trials with EEG artifacts identified by automatic artifact removal and manual inspection also were discarded from behavioral analysis such that the very same epochs/trials were used for the analysis of behavior and SSVEP amplitudes. This was important to remove epochs where participants’ eye gaze moves toward the relevant cued stream.

For the exploratory P300 analysis, one participant (AVGP) was excluded due to having had no correct responses (hits) in at least one condition out of the eight. For the ERN analysis, five participants were excluded (4 AVGPs, 1 NVGP), because they did not have any false alarms in at least one condition. The final sample sizes consisted of 67 participants for the P300 and 63 participants for the ERN analyses.

##### EEG

**Fig. 2:**
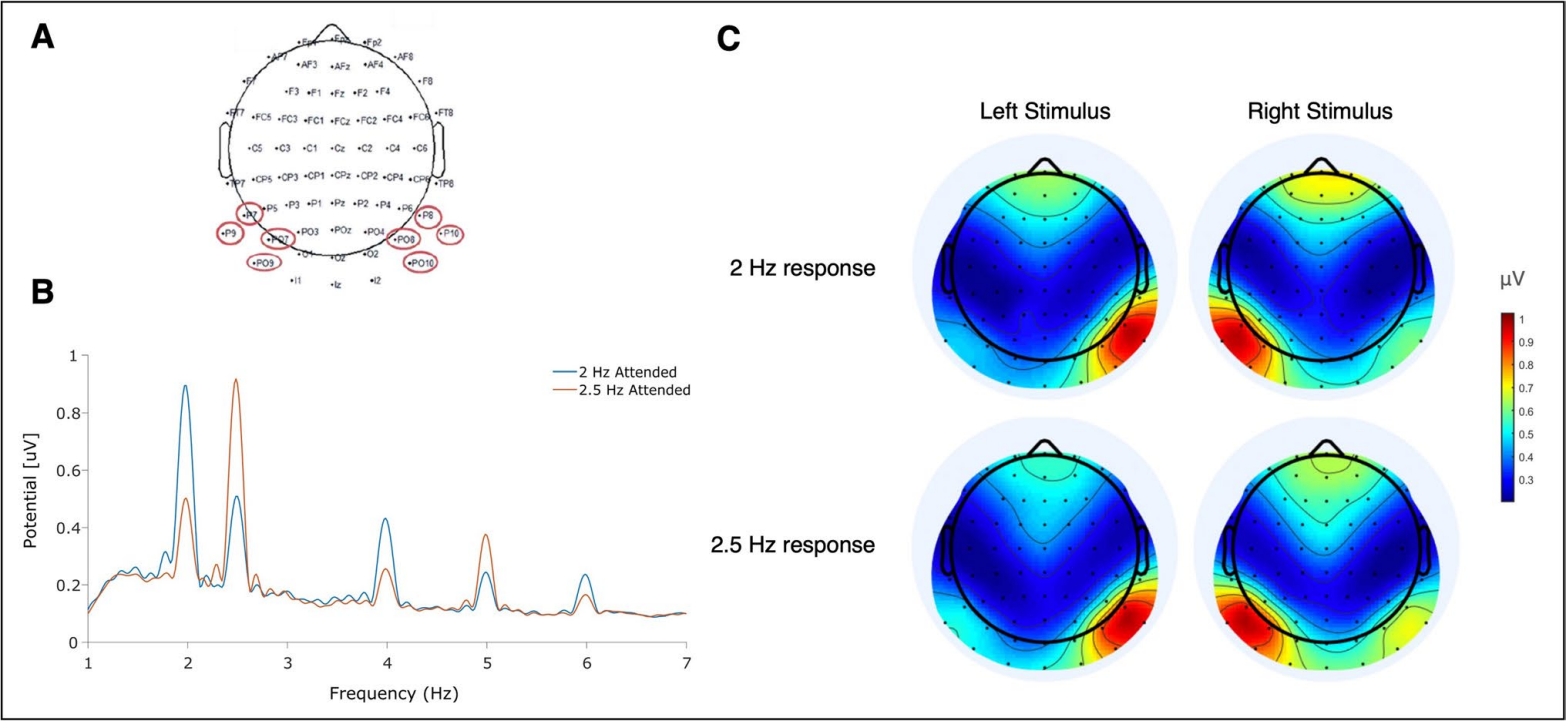
SSVEP topographies, time course and spectra. Panel **A**: Cluster of the four left and right parieto-occipital electrodes used in all analyses (circled in red). Panel **B**: Grand-average power spectrum obtained by a Fourier transform of SSVEP waveforms for each condition averaged across the electrode cluster described in A. Peaks correspond to the respective stimulation frequencies and their harmonics. Panel **C**: Spline-interpolated isocontour voltage maps of SSVEP amplitudes for both stimulation frequencies (averaged over all experimental conditions, separately for left and right stimuli).

SSVEP amplitudes were extracted using a Fast Fourier Transformation (FFT) applied to the averages of trials within each of the eight task conditions at either of the two presentation frequencies (2.0 Hz or 2.5 Hz). A 7-s time window was used for this, removing the first and last 500 ms of the 8-s stimulus sequences. The first 500 ms of the sequence were removed to allow the visual evoked potentials enough time to become a steady periodic response. The last 500 ms were additionally removed to exclude eye movements or other movement artifacts, because these showed a tendency to occur more frequently toward the end of trials. The 7-s segments were re-referenced to the average of all electrodes. Following the FFT, the SSVEP amplitudes of each subject and frequency (2.0 Hz or 2.5 Hz) were rescaled by dividing by the mean amplitude of all eight conditions (Andersen et al., 2011). The computed SSVEP amplitudes were collapsed across frequencies after verifying that frequency impacted neither SSVEP amplitudes nor accuracy. The amplitudes were subjected to a between-group repeated measures ANOVA with the following factors (left vs. right, positive target emotion vs. negative target emotion, AVGP vs. NVGP, attended vs. unattended). We also considered the data collection phase as an additional factor. Grand mean topographical maps were plotted to show peaks at lateral parietal-occipital locations at both frequencies and in both groups (Fig. 2A). Following the visual inspection of these topographies, two clusters of four electrodes were selected and averages were computed over these electrodes within each hemisphere: P7, PO7, P9, PO9 (left hemisphere); P8, PO8, P10, PO10 (right hemisphere). Extracted amplitudes to attended and unattended streams were used to calculate the grand averages of SSVEP amplitudes for respectively the attended and unattended conditions in each group. The SSVEP peak amplitude topographies show the expected pattern of SSVEP responses to attended and unattended streams and thereby frequencies, whereby peaks at 2.0 Hz and 2.5 Hz were identifiable (Fig. 2B, C).

##### Statistics

Data were analyzed using R. We used ANOVA comparisons to test the main effects and interactions. We reported additional Student’s t-tests and Tukey’s tests for post-hoc comparisons based on the ANOVA results. Pearson correlations are used and an alpha of .05 was used to report statistical significance. Greenhouse-Geisser correction was applied to degrees of freedom to correct for nonsphericity where appropriate. In line with our pre-registered methods, we also conducted Bayesian ANOVAs and reported the Bayes’ Factor assessing the amount of evidence in favor of H1 over H0 (BF10). Importantly, all Bayesian analyses are performed without any prior in order to obtain the more conservative and least biased estimates, considering that the literature makes opposite predictions regarding the relationship between video gameplay and performance in the attention-demanding emotion discrimination task (literature on violent games predicts impaired emotion processing, whereas literature on action games predicts improved perceptual abilities but no difference in emotion processing between action game players and nonplayers).

## Results

### Behavioral results

**Fig. 3:**
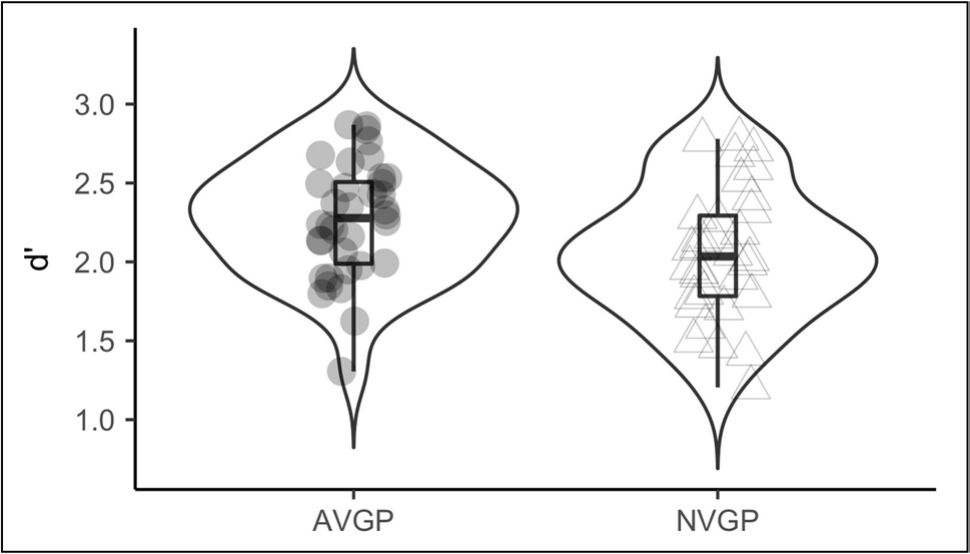
Behavioural performance in attention-demanding emotion discrimination task. AVGPs (*n* = 36) outperform NVGPs (*n* = 32) in emotion discrimination

Sensitivity index, *d’* was used to assess the accuracy of emotion discrimination in the attention-demanding emotion discrimination task. We ran a repeated-measures mixed-model ANOVA with repeated measures factors of side (left, right) and target (fear/disgust or happy/surprise). Group (AVGP, NVGP) and data collection phase (phase 1, phase 2) were assigned as between-subject factors. As expected, we found a main effect of Group, *F*(1,64) = 4.47, *p* = 0.038, *η*^*2*^ = 0.050 (Fig. 3). In line with our hypothesis, AVGPs outperformed NVGPs at an attention-demanding emotion discrimination task (AVGPs: *M* = 2.25, 95% CI = 2.12-2.38; NVGPs: *M* = 2.05, 95% CI = 1.91-2.19; *t*(64) = 2.12, *p* = 0.038, Cohen’s d = 0.54).

To investigate whether the reported differences were due to differences in emotion recognition ability, all participants were assessed on their ability to categorize facial emotional stimuli by emotion type, intensity, and valence with no time limit. The emotion recognition accuracy did not differ between groups, *F*(1,64) = 0.66, *p* = 0.419, *η*^*2*^ = 0.003. The evidence toward an absence of a main effect of group was strong with a BF10 = 0.16. This indicates that the group difference in emotion discrimination in the SSVEP task is unlikely to result from a difference in facial emotion recognition when attention is not explicitly manipulated. There was a main effect of target emotion, *F*(2.42,154.56) = 32.90, *p* < 0.001, *η*^*2*^ = 0.279, driven by higher accuracy for recognizing happy faces compared with emotions of surprise, anger, and disgust (all *p’s* < 0.001).

#### EEG: SSVEP Amplitudes

We ran a repeated-measures mixed-model ANOVA with attention (attended, unattended), side (left, right), and target emotions (fear/disgust or happy/surprise) as repeated measures and Group (AVGP, NVGP) and data collection phase (phase 1, phase 2) as between-subject factors. The analysis revealed two weak interactions with data collection phase: a data collection phase by target emotions interaction, *F*(1,64) = 5.42, *p* = 0.023, *η*^*2*^ = 0.012 and a 3 way interaction between data collection phase, attention, and side *F*(1,64) = 7.84, *p* = 0.006, *η*^*2*^ = 0.008. Because data collection did not interact with our primary factors of interest—group and attention, these effects will not be discussed further.

**Fig. 4:**
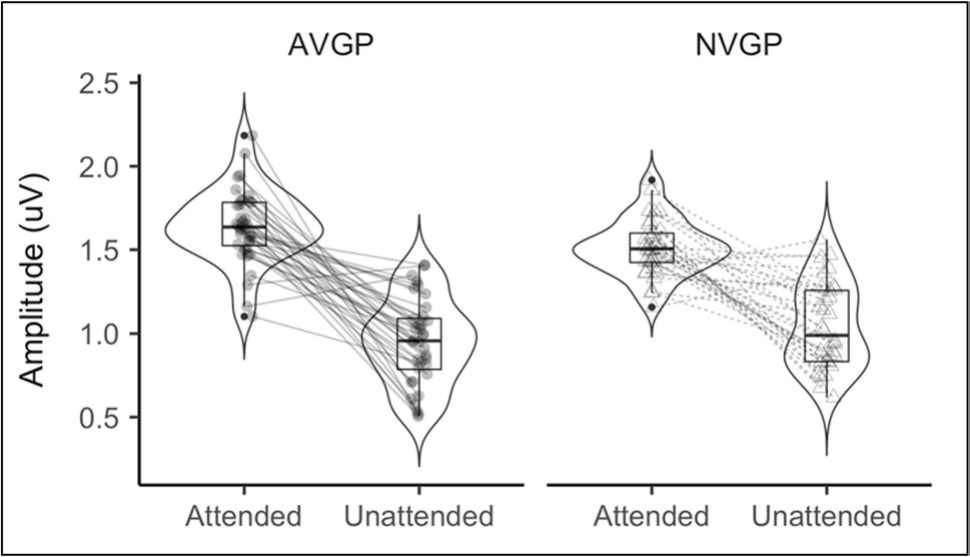
SSVEP amplitudes for attended and unattended stimuli by group. Attended streams consistently show greater amplitude

The expected effect of attention, *F*(1,64) = 146.13, *p* < 0.001, *η*^*2*^ = 0.463 was driven by higher amplitudes to attended stimuli, compared with unattended stimuli. Further registered comparison revealed that this effect was present in both AVGPs, *t*(35) = 10.41, *p* < 0.001, *d* = 2.82, and NVGPs, *t*(32) = 6.79, *p* < 0.001, *d* = 2.13. Importantly, the main effect of attention interacted with group, *F*(1,64) = 5.00, *p* = 0.029, *η*^*2*^ = 0.029. A group difference was observed for the attended stream, *t*(66) = 2.54, *p* = 0.013, *d* = 0.63, whereas no group difference was found for the unattended stream, *t*(66) = −1.36, *p* = 0.180, *d* = −0.37 (Fig. 4).

The main effect of side *F*(1,64) = 15.93, *p* < 0.001, *η*^*2*^ = 0.030, was driven by higher amplitudes for stimuli presented on the left (i.e., processed by the right hemisphere) compared with stimuli presented on the right (processed by the left hemisphere). The main effect of target emotions, *F*(1,64) = 6.24, *p* < 0.001, *η*^*2*^ = 0.014 was driven by higher amplitudes for streams for which anger and disgust were the target emotions (i.e., mostly positive happy and/or surprised distractor faces were viewed) compared to streams for which happy and surprise were the target emotions (i.e., mostly negative angry and/or disgusted distractor faces were viewed).

An unexpected triple interaction between group, attention and side was found*, F*(1,64) = 11.87, *p* < 0.001, *η*^*2*^ = 0.009. This suggests a different pattern of attention by side interaction in the AVGP and NVGP groups. AVGPs showed larger amplitudes to left stimuli as compared to right stimuli in the unattended stream, *t*(35) = 4.70, *p* < 0.001, *d* = 0.73, whereas no difference was observed between left and right stimuli in the attended stream, *t*(35) = 0.66, *p* = 0.51, *d* = 0.09. NVGPs showed an opposite pattern with no difference between left and right stimuli in the unattended stream, *t*(31) = 1.51, *p* = 0.14, *d* = 0.25, whereas larger amplitudes were observed to left stimuli as compared to right stimuli in the attended stream, *t*(31) = 2.69, *p* = 0.009, *d* = 0.51.

#### Attention control tasks

We also measured attention control with standard measures of attention on a subset of the total sample size, collected in the second phase of data collection only (*n* = 38). For the MOT, correctness of responses decreased with target number (main effect of target number: *F*(3.118, 115.13) = 54.25, *p* < 0.001, *η*^*2*^ = 0.446). However, no group differences were found, *F*(1,36) = 0.043, *p* = 0.836, *η*^*2*^ = 0.001, with comparable mean accuracy between AVGP (*M* = 0.829, *SD* = 0.162) and NVGP (*M* = 0.823, *SD* = 0.161) (Supplemental Material 1). For the UFOV measures, display duration was numerically smaller for AVGP than NVGP as expected (*M* = 37.33, *SD* = 41.7) and NVGP (*M* = 62.76, *SD* = 51.1), but this effect was not significant, *t*(1, 32.88) = −1.68, *p* = 0.10, *d* = 0.54 with weak evidence for the effect (BF10 = 0.97) (Supplemental Material 2).

#### Relationship between Behavior and SSVEP markers of attention—Exploratory analyses

Using d′ as a measure of behavior, correlation analysis indicated that SSVEP amplitudes to the attended stream were significantly correlated with d′, r(66) = 0.32, *p* = 0.0073. The direction of this relationship appears to hold, with a similar effect size, although the correlation is not statistically significant when looking at AVGP (r(34) = 0.27, *p* = 0.15) and NVGPs (r(30) = 0.27, *p* = 0.11) separately. Additionally, the difference between attended and unattended SSVEP amplitudes marginally correlated with the behavioral metric of d', r(66) = 0.23, *p* = 0.054. Here, the effect size of that relationship was numerically greater for AVGPs (r(34) = 0.22, *p* = 0.21) than NVGPs ( r(30) = 0.12, *p* = 0.5).

## Discussion

The present study asked whether the attentional benefits previously documented in AVGPs when processing simple, non-emotional stimuli extend to complex emotional stimuli, such as facial emotions. AVGPs outperformed NVGPs in an attention-demanding facial emotion discrimination task. This difference was not accounted for by a group difference in the ability to recognize facial emotions. SSVEPs revealed higher attentional modulation in AVGPs compared with NVGPs, in line with previous reports of greater attentional control in AVGPs. No group differences were observed in the more classical ERP components of P300 associated with target detection and ERN associated with the error made during the task (Supplement Material 3). This enhanced attentional processing of facial emotions in AVGPs was quantified in a perceptually and attentionally demanding task, departing from previous work using emotional stimuli.

The novel emotion discrimination task revealed a group difference between AVGPs and NVGPs in the ability to identify target emotions in a rapidly and peripherally presented stream of facial emotions. This difference was observed despite the similar facial emotion recognition abilities between the two groups. Indeed, the AVGPs and NVGPs performed equally well when asked to correctly identify facial emotions from single faces presented centrally and without any time constraint. Both groups recognized happy faces with higher accuracy. This happy face advantage has been reported previously using various datasets of emotional stimuli (Calder et al., 2000; Calvo and Beltrán, 2013; Leppänen and Hietanen, 2004; Palermo and Coltheart, 2004).

The finding that AVGPs outperform NVGPs in an attentionally demanding emotional discrimination task (i.e., under high attentional load) contrasts with previous work from our group and others in which AVGPs and NVGPs showed comparable performance in a simple (i.e., low attentional load) task of facial emotion discrimination (Bailey and West, 2013; Pichon et al., 2021). As these tasks involved emotion discrimination but differed in attentional load for this paradigm, the AVGP advantage observed here may arise from group differences in attention rather than differences in emotion processing per se. In line with this view, similar mental representations of emotional faces were observed in AVGs and NVGPs as assessed by reverse inference techniques (Pichon et al., 2021). However, further studies contrasting emotional processing under high compared with low load in AVGPs and NVGPs would be important to confirm this interpretation.

The differences observed between AVGPs and NVGPs indicate that attentional advantage in AVGP extends to emotional stimuli, likely through a balance of suppression of irrelevant stimuli and enhancement of relevant stimuli. Previous studies using non-emotional stimuli have often documented suppression of distractors as a mechanism for this advantage rather than enhancement of the attended stream (Krishnan et al., 2013; Mishra et al., 2011). In contrast, the present study indicates that the attentional advantage of AVGPs emerge more reliably from attentional enhancement of the attended stream. This discrepancy is consistent with the idea that emotional stimuli readily attract attention to their spatial location rendering suppression possibly more difficult (Pourtois and Vuilleumier, 2006; Vuilleumier, 2005; Vuilleumier and Huang, 2009). While the attentional pull of emotional stimuli is known to be more marked when attentional load is low (Pessoa, 2005; Pessoa et al., 2002), it may remain that, in the present design, selecting emotional content amongst other emotional content renders suppression of the task-irrelevant stream of emotional faces harder. Alternatively, the distinction within the attended stream between different facial emotion may be more effortful than that of finding digits among letters. This may lead to greater enhancement of the task-relevant stream. However, distinctions between attentional enhancement and suppression are hard to make in the absence of a neutral comparison level (Wöstmann et al., 2022). Further studies are needed to confirm whether attentional modulation mechanisms differ between emotional and nonemotional stimuli. Whether these differences in attention mechanisms are driven by the emotional nature of the stimuli or the selection difficulty of the task will require future studies.

To assess group differences in attentional resource allocation toward emotional stimuli, we used SSVEPs as a highly sensitive discrimination measure of attentional modulation. Given the use of facial emotional stimuli presented peripherally, slower than standard presentation rates were used (2.0 Hz and 2.5 Hz). Indeed, initial studies on similar divided spatial attention paradigms for nonfacial emotional stimuli used higher presentation frequencies—in the range of 8.6 Hz and 12.0Hz (Morgan et al., 1996; Norcia et al., 2015; Zhu et al., 2016). Slow presentation frequencies for emotional faces have been used before to compensate for the difficulty of task processing (Dzhelyova et al., 2016; Rossion and Boremanse, 2011; Zhu et al., 2016). In our case, they were further lowered given the lateralized presentation at 5.3° eccentricity (each face subtending a 6.75° × 7.4° visual angle) while maintaining central fixation. This presentation method elicited reliable SSVEP responses with high SNR to both attended and unattended streams providing a neural measure of attentional modulation.

For a subset of the participants, two attentional control tasks were administered at the end of the protocol. The expected group difference in multiple object tracking was not observed. The reason for this lack of difference remains unclear. In the case of the UFOV, another measure of attentional control that heavily loads on divided attention, we also did not find a significant group difference. However, AVGPs numerically outperformed NVGPs as they required about half the display time that NVGPs did to reach the same level of performance. The effect size of 0.54 matches the medium effect reported in the literature for cross-sectional differences in top-down attention between these two groups (see Bediou et al., 2018—Hedges’ g of 0.625 with a 95% CI between 0.494 and 0.756). Although stronger results would have been welcomed to externally validate the group selection and their attentional differences, the attentional task results are consistent with AVGPs having, albeit weakly, greater attentional control skills than NVGPs (Green and Bavelier, 2003).

The present study also highlights the main effect of attended side with higher SSVEP amplitudes for emotional faces presented on the left and thus processed by the right hemisphere. This observation is in line with the literature reporting a right-hemispheric bias for face processing (Grand et al., 2003; Kanwisher et al., 1997; Rossion et al., 2000). Furthermore, higher SSVEP amplitudes were noted when anger and disgust were the target emotions (among a majority of happy and surprised faces) compared with when happy and surprised faces were the target (among a majority of anger and disgusted faces). The interpretation of this effect is not straightforward, as physical stimuli and attentional demands are confounded here. Happy and surprised faces may elicit larger amplitudes irrespective of tasks and attention (e.g., due to physical stimulus properties). Alternatively, this could arise from a greater attentional demand to detect negative targets or a greater demand to suppress (or disengage) from positive distractors. The latter case is supported by the recognition advantage for happy emotions, shown in this study alongside many previous reports (Calvo et al., 2010; Calvo and Beltrán, 2013). This happy-face advantage may drive the increased amplitudes compared with when angry and disgusted faces represent the majority of stimuli.

Interestingly, the main attentional difference between groups was found independently of the emotional status of the target (positive or negative), indicating that the greater attentional modulation in AVGPs is similarly robust across positive and negative valence emotional signals. Previous work on violent video games and emotion processing has yielded inconsistent results regarding a possible reduced happy face advantage in violent video game players (Kirsh and Mounts, 2007) or changes in the processing of negative emotions (Diaz et al., 2016; Miedzobrodzka et al., 2021). The present work contributes evidence that AVGPs do not differ from NVGPs in emotion identification per se, but rather show enhanced emotion discrimination in attention-demanding conditions.

An unexpected and weak triple interaction between group, attention, and side also was observed. Greater SSVEP amplitudes to unattended faces when presented in the left visual field (right FFA) rather than the right visual field were observed in AVGPs. This may be accounted for by more automated access to irrelevant information in this group, in line with the hypothesized greater attentional resources in AVGPs. In line with the Load theory (Lavie et al., 2004) at low and intermediate loads, AVGPs have been shown to process task-irrelevant information to a greater extent than NVGPs, without resulting in a loss of performance (Bavelier et al., 2012; Dye et al., 2009). In contrast, NVGPs displayed greater SSVEP amplitudes for attended, left visual field (right FFA) faces compared with attended right visual field (left FFA) faces. As for the brain regions where attention was quantified, in line with previous work (Adamian et al., 2019; Andersen et al., 2011; Antonov et al., 2020), both SSVEP responses and their modulation by attention were maximal over bilateral parieto-occipital areas (Fig. 2C), supporting the idea that attentional modulation occurred in early visual areas, rather than in later and more anterior areas of the processing stream (e.g., parietal areas).

In sum, this SSVEP study presents a novel, attention-demanding facial emotion discrimination task that allows monitoring of neural mediators for attended and unattended facial emotions. We confirmed a right hemisphere bias for facial processing as well as a happy face advantage. Given our aim to evaluate if the greater attentional control described in AVGPs using nonaffective stimuli generalizes to affective stimuli we compared AVGP and NVGP responses. We found greater attentional modulation in AVGPs than NVGPs as facial emotions required discrimination amongst distractors. This group effect had a medium effect size (*d* = 0.54 for the behavioral difference, *d* = 0.63 for the attended amplitude difference). This group difference is striking given that several reports, including ours here, report identical facial emotion recognition skills under low attentional demands. We, therefore, highlight attentional processing differences in AVGPs and NVGPs and the generalization of attentional benefits beyond perceptual and cognitive processes in AVGPs.

## Supplementary Information

ESM 1 (DOCX 331 kb)

## Data Availability

Pre-registration for this study can be found on https://osf.io/y2w6u.
