# Supplementary material for: Attentional modulation as a mechanism for enhanced facial emotion discrimination: The case of action video game players
Source: Cogn Affect Behav Neurosci. 2023 Jan 20;23(2):276–89. doi: 10.3758/s13415-022-01055-3 (PMC10050043; doi:10.3758/s13415-022-01055-3)
Supplement: Supplementary file 1 — (DOCX 331 kb) [file 13415_2022_1055_MOESM1_ESM.docx]

**Supplementary Information**

**Supplemental Material 1, Figure 1:**

**
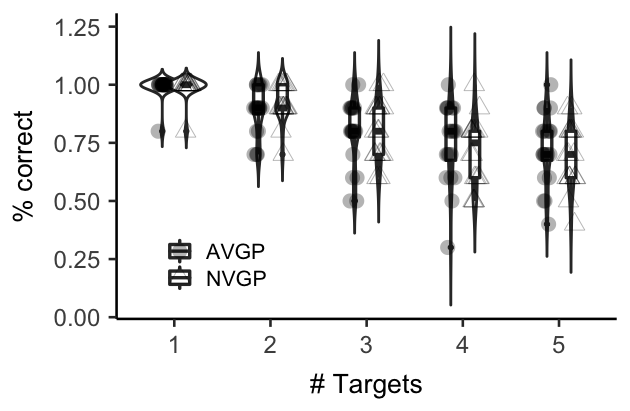
**

**Supplemental Material 2, Figure 2:**

**
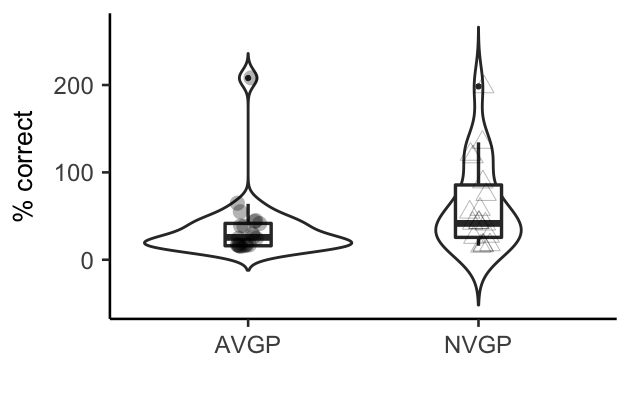
**

**Supplemental Material 3**

Exploratory P300 & ERN results

As described in our pre-registered methods, the attention-demanding emotion discrimination task presented in this work was designed to elicit SSVEP, that is, naturally occurring oscillatory responses to continuously presented visual stimuli flickering at specific frequencies. Critically, SSVEP provide a sensitive neural measure of attentional modulations within the visual pathway (Andersen & Muller, 2010). Neural responses to attended (relevant) and unattended (irrelevant) visual streams can be quantified separately through frequency-tagging over posterior electrodes (e.g., Toffanin et al., 2009). As such, SSVEP allow indexing of the enhancement of attended stimuli and the suppression of unattended stimuli. This captures the push-pull mechanism of attention described along the visual pathway (Desimone & Duncan, 1995; Reynolds & Heeger, 2009). Here we present the event-related potentials of P300 and ERN which in contrast index more decisional processes, reflecting selection of infrequent transient target events.

The P300 is a centro-parietal component evoked in the process of decision making. It is typically larger in response to correctly detected targets compared to missed ones (Hillyard et al., 1971). Its latency varies closely with the reaction time of participants (McCarthy & Donchin, 1981). While there is still disagreement as to when in the decision process the P300 emerges (Nieuwenhuis et al., 2005; Polich, 2007), it is accepted that it is far from a unitary component. For example, in designs optimized to investigate P300, a common distinction is made between the P3a, thought to originate from stimulus-driven disruption of frontal attention engagement during task processing, and the P3b, proposed to index temporo–parietal mechanisms of memory. Given our design was optimized for SSVEPs and not ERPs (lack of an informative tight baseline), here we consider the whole P300 complex.

The P300 component in the ERPs time-locked to targets was quantified over five parieto-occipital sites (P1, P2, POz, Pz, CPz) over the 550ms - 800ms time window. This window included the peak grand-averaged waveforms with respect to a pre-stimulus baseline (-250ms to 0ms). P300 amplitudes were quantified in the -250ms to +1000ms interval surrounding the peak grand-averaged waveforms Following the previous findings (Mishra et al., 2011), we asked whether the P300 amplitude difference between targets and non-targets may be larger in AVGPs compared to NVGPs. To this end, we used a repeated measure ANOVA with group and stimulus type. A greater stimulus-locked P300 response to targets was observed compared to non-targets F(1,65) = 195.08, p <.001, η^2^ = .499. There was also a marginal and weak effect of group F(1,65) = 2.94, p = .091, η^2^ = .020 which reflected smaller amplitudes to both targets and non-targets in AVGPs vs NVGPs. A marginal interaction between data collection phase and emotion was also found, F(1,65) = 4.32, p = .042, η^2^ = .008 (Supplemental Material 3, Figure 3).

The error-related negativity (ERN) is another electrophysiological marker related to decision-making which is time-locked to responses and is larger to errors than correct responses. The ERN generally occurs over frontocentral electrodes about 30-80 ms after an erroneous response (van Veen & Carter, 2006). Although our study wasn’t specifically designed to investigate the ERN, we re-epoched from -1000ms to +500ms after correct and incorrect responses. The baseline was set at -1000ms to 0ms. We quantified the ERN as the negative fronto-central deflection occurring within +15ms and +55ms following an erroneous response (i.e., false alarm). The ERN was quantified as the mean amplitude at FCz within this time window. The error-related negativity (ERN) elicited by false alarms was greater compared to hits *F*(1,61) = 30.31, *p* < .001, *η^2^* = .038. An unexpected triple interaction of data collection phase, emotion and errors made was also observed *F*(1,61) = 5.23, *p*  =  .02, *η^2^* = .026.  No main effect of group was found for the ERN, *F*(1,61) = 0.230, *p* = .633, *η^2^* = .0.003, with a BF10 of 0.18 and  no significant interactions with the group factor were found.

**
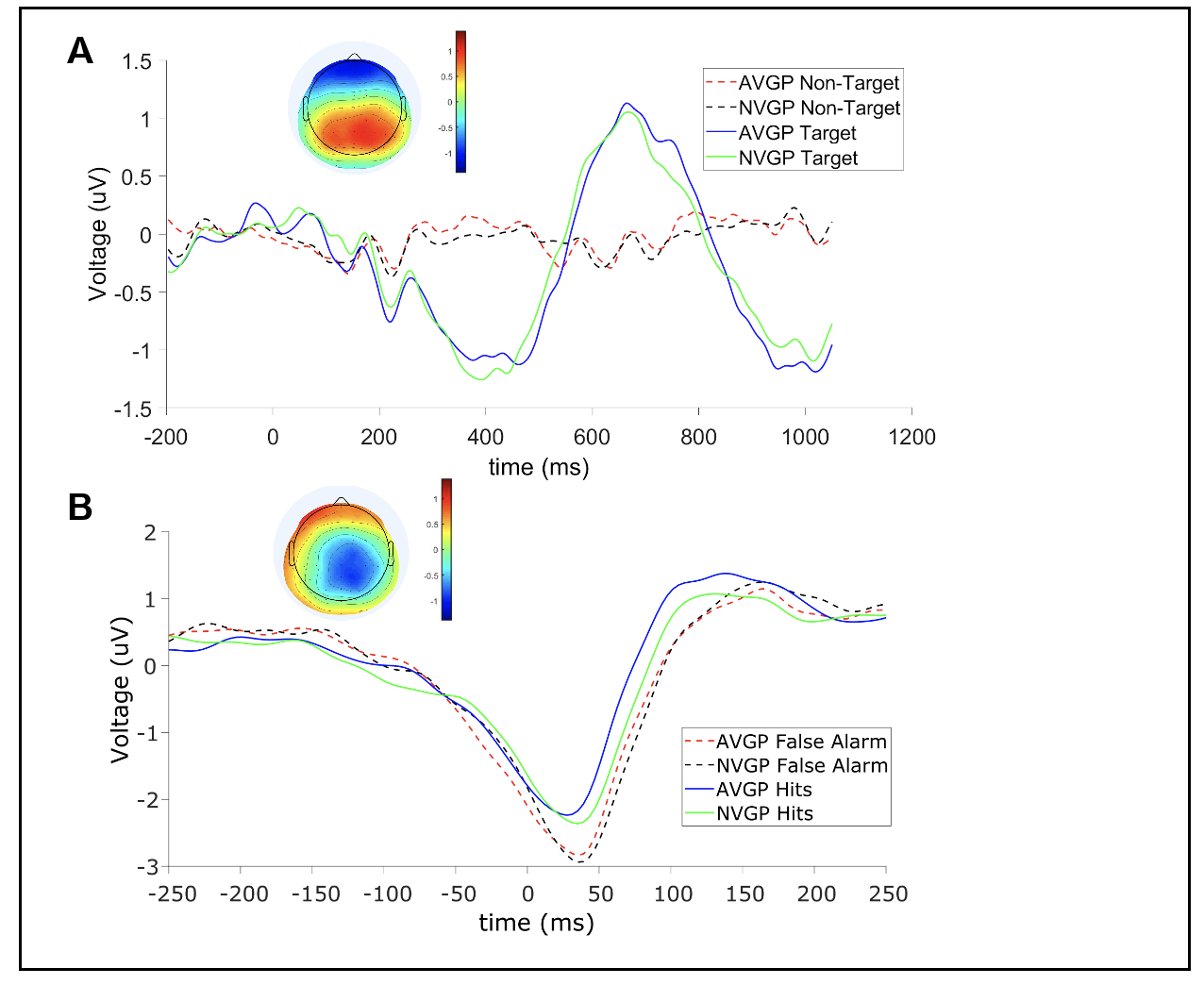
**

**Figure 3:** A) P300 for channels P1, Poz, Pz, CPz and P2 with corresponding topography from targets minus nontargets within 550ms-800ms. B) ERN plot for channel Fcz with corresponding topography from FAs minus hits within 15ms - 55ms.

**References**

Andersen, S. K., & Muller, M. M. (2010). Behavioral performance follows the time course of neural facilitation and suppression during cued shifts of feature-selective attention. *Proceedings of the National Academy of Sciences*, *107*(31), 13878–13882. https://doi.org/10.1073/pnas.1002436107

Desimone, R., & Duncan, J. (1995). Neural Mechanisms of Selective Visual Attention. *Annual Review of Neuroscience*, *18*(1), 193–222. https://doi.org/10.1146/annurev.ne.18.030195.001205

Hillyard, S. A., Squires, K. C., Bauer, J. W., & Lindsay, P. H. (1971). Evoked Potential Correlates of Auditory Signal Detection. *Science*, *172*(3990), 1357–1360. https://doi.org/10.1126/science.172.3990.1357

McCarthy, G., & Donchin, E. (1981). A Metric for Thought: A Comparison of P300 Latency and Reaction Time. *Science*, *211*(4477), 77–80. https://doi.org/10.1126/science.7444452

Mishra, J., Zinni, M., Bavelier, D., & Hillyard, S. A. (2011). Neural Basis of Superior Performance of Action Videogame Players in an Attention-Demanding Task. *Journal of Neuroscience*, *31*(3), 992–998. https://doi.org/10.1523/JNEUROSCI.4834-10.2011

Nieuwenhuis, S., Aston-Jones, G., & Cohen, J. D. (2005). Decision making, the P3, and the locus coeruleus—Norepinephrine system. *Psychological Bulletin*, *131*(4), 510–532. https://doi.org/10.1037/0033-2909.131.4.510

Polich, J. (2007). Updating P300: An integrative theory of P3a and P3b. *Clinical Neurophysiology*, *118*(10), 2128–2148. https://doi.org/10.1016/j.clinph.2007.04.019

Reynolds, J. H., & Heeger, D. J. (2009). The Normalization Model of Attention. *Neuron*, *61*(2), 168–185. https://doi.org/10.1016/j.neuron.2009.01.002

Toffanin, P., de Jong, R., Johnson, A., & Martens, S. (2009). Using frequency tagging to quantify attentional deployment in a visual divided attention task. *International Journal of Psychophysiology*, *72*(3), 289–298. https://doi.org/10.1016/j.ijpsycho.2009.01.006

van Veen, V., & Carter, C. S. (2006). Error Detection, Correction, and Prevention in the Brain: A Brief Review of Data and Theories. *Clinical EEG and Neuroscience*, *37*(4), 330–335. https://doi.org/10.1177/155005940603700411
